# Prevalence of selected Shiga toxin-producing Escherichia coli vaccine antigen genes among two geographically distinct ruminant populations

**DOI:** 10.1099/jmm.0.002132

**Published:** 2026-03-19

**Authors:** Conor Quinn, Rhys Bruce, Joanne Cosgrave, Laura Sala-Comorera, Niamh Martin, Catherine M. Burgess, Elena-Alexandra Alexa, Catherine McAloon, Susanna Frost, Emmanuel Okello, Geraldine Duffy, Sharif S. Aly, Siobhán McClean

**Affiliations:** 1 School of Biomolecular and Biomedical Sciences, University College Dublin, Belfield, Dublin 4, Ireland; 2 UCD Conway Institute, University College Dublin, Belfield, Dublin 4, Ireland; 3 Food Safety Department, Teagasc Food Research Centre, Ashtown, Dublin 15, Ireland; 4 School of Veterinary Medicine, University College Dublin, Belfield, Dublin 4, Ireland; 5 Department of Clinical Microbiology, Tallaght University Hospital, Dublin, Ireland; 6 Department of Population Health & Reproduction, School of Veterinary Medicine, University of California, University of California Davis, Tulare, CA, USA; 7 Veterinary Medicine Teaching and Research Center, School of Veterinary Medicine, University of California Davis, Tulare, CA, USA

**Keywords:** bovine, One Health, ovine, real-time PCR, Shiga toxin-producing *Escherichia coli* (STEC), vaccine antigen prevalence

## Abstract

**Introduction.** Shiga toxin-producing *Escherichia coli* (STEC) are a group of bacteria that cause severe bloody diarrhoea and haemolytic uraemic syndrome and are associated with neurological complications and potentially life-threatening infections. Children under five and elderly individuals are particularly vulnerable to STEC infections. The majority of STEC infections have been traced back directly or indirectly to ruminants, and there is an unmet need for a vaccine to protect these vulnerable cohorts.

**Gap Statement.** We previously identified a series of STEC proteins that are involved in attachment to human gastrointestinal epithelial cells, some of which are protective in a murine challenge model. However, the prevalence of the genes in dairy herds was unknown.

**Aim.** Due to zoonotic transmission, the development of a successful human vaccine relies on any protective vaccine antigens being prevalent in ruminant herds in different geographical locations. We aimed to examine the potential geographical differences in antigen gene prevalence.

**Methodology.** Faecal isolates collected from a dairy herd in CA, USA, and isolated from dairy cows in Ireland were analysed by quantitative PCR. Bioinformatic analysis of previously sequenced isolates was also performed.

**Results.** Four antigen genes were highly prevalent among STEC shed from cattle in a Californian dairy herd (>95.7%). Genes encoding FkpA, YiaF and GlnH were also highly prevalent in faecal samples from cows sampled in Ireland (93%). The point prevalence of *terD* among cattle in the Irish cohort appeared low relative to Californian isolates, but this is most likely due to the absence of tellurite in isolation media used in the former. Expansion of the study to examine Irish bovine and ovine Whole genome sequencing datasets increased the detection of *terD* in Irish isolates.

**Conclusion.** Overall, three antigen genes are prevalent in both geographically distinct cohorts. Discrepancies in *terD* gene prevalence are likely due to the use of selective agars in isolation protocols.

## Data Summary

Short-read sequence files and completed assemblies of ovine isolates were submitted to the European Nucleotide Archive under the study accession number PRJEB46014 and to NCBI under the BioProject accession number PRJNA744868, while those of the bovine isolates were uploaded under the BioProject accession number PRJNA912741. The specific accession numbers of the bovine isolates are SAMN32253146, SAMN32253145, SAMN32253144, SAMN32253143, SAMN32253142, SAMN32253141, SAMN32253140, SAMN32253139, SAMN32253138, SAMN32253137, SAMN32253136 and SAMN32253135.

## Introduction

Shiga toxin-producing *Escherichia coli* (STEC), also known as verocytotoxigenic *E. coli*, are a group of strains of *E. coli* that cause severe bloody diarrhoea. The incidence and severity are greatest in children under five, and the infection can also cause haemolytic uraemic syndrome (HUS), which is the main cause of kidney failure in this cohort of children [1,2]. HUS can also cause life-long complications, including seizures and blindness. STEC O157:H7 is the predominant serotype associated with outbreaks, but non-O157 STEC strains are also important causes of acute diarrhoea, dysentery and HUS [3,4]. In 2021, there were 6,534 STEC cases reported by 30 countries in the European Union (EU), with a notification rate of 2.2 cases per 100,000 population, and its incidence is increasing, which may be due in part to advances in molecular diagnostics [4,6]. The rate varies across the EU and is second highest in Ireland with 15.8 notifications per 100,000 which is five times the EU average. The highest rate is currently in Denmark at 24.12 notifications per 100,000 [7]. This report also noted that a high proportion of HUS cases in the EU occur due to non-O157 serogroups, with O26 cases accounting for 40% of HUS cases in 2023. Indeed, an O26:H11 STEC outbreak in Italy in 2013 resulted in 20 confirmed HUS cases in children aged between 11 and 78 months, 2 of whom were reported to have severe neurological sequelae in the subsequent 18 months [8]. Moreover, the US CDC estimates that STEC O157 and non-O157 strains account for about 266,000 illnesses and 3,670 hospitalizations annually [9]. Because the proportion of STEC patients, particularly children, experiencing severe symptoms is high, there is an unmet need for a vaccine.

STEC is the only pathogenic group of *E. coli* that has a definite animal origin, and cattle are the predominant reservoir. To date, vaccine development has been limited to a small number of virulence factors and has focussed predominantly on the ruminant reservoir [10,13]. Human STEC vaccine development has also been largely restricted to these virulence factors, including Stx antigens, intimin and EspA. However, for STEC to colonize the human gut, it is likely to require the use of combinations of adhesin proteins [14,18]. We previously identified a series of STEC proteins that were involved in attachment to human gastrointestinal cell lines Caco-2 and HT29 cells and that were absent in the commensal control strain, HS [19]. Preclinical studies are ongoing, but the results to date are promising; for example, GlnH reduces bacterial colonization in immunized mice by 18-fold [19]. Other proteins identified as being involved in host cell attachment were FkpA [19], and more recently, a putative tellurium resistance protein TerD and an uncharacterized protein YiaF were also identified as being involved in attachment to HT29 cells. However, for any prophylactic vaccine to be useful, the antigens need to be expressed in isolates across the globe. The overall aim of this study was to examine the prevalence of the identified antigens in STEC isolated from ruminants to determine whether these antigens are conserved across two geographically distinct populations (Ireland and California), specifically to evaluate their potential as vaccine candidates. The prevalence of four selected antigen genes in STEC shed from ruminants was assessed to identify candidates that might offer coverage across STEC serotypes in different regions. The analysis was supplemented by an additional *in silico* analysis.

## Methods

### PCR analysis of clinical paediatric STEC-confirmed isolates

At the time of the study, five anonymized STEC-confirmed isolates from hospitalized paediatric patients were obtained from the National Children’s Hospital, Tallaght, for an initial investigation of the presence of the four antigen genes of interest in STEC patients. Colony PCR was performed by boiling a single colony of each isolate at 95 °C for 10 min. Each PCR reaction master mix contained HotStart Taq Master Mix taq polymerase (25 µl), sterile water (15 µl) and the appropriate forward and reverse primers (Table S1, available in the online Supplementary Material, 2.5 μl of each), which was added to PCR tubes with the appropriate DNA template (5 µl). The following amplification conditions were used: 95 °C denaturation, 58 °C annealing and 72 °C synthesis for 38 cycles, before analysing samples on 2% agarose gels at 100 V for 1 h.

### Faecal and serum collection, processing and DNA isolation from the Californian herd population

The study protocols and procedures employed were ethically reviewed and approved by the University of California Davis’ Institutional Animal Care and Use Committee (protocol number 22017). Sample size was determined as 89 from a population of 100 dairy cows required to achieve 80.911% power to detect a difference (P1-P0) of 0.0500 using a two-sided exact test with a significance level (alpha) of 0.05. Given the potential for DNA extraction failure and issues in shipping samples, an additional 50% was added, resulting in 130 cows. Thus, a total of 130 dairy cows from a Californian herd population were selected at random from two separate herd pens, and faecal samples were collected from each cow within 1 day. Faecal samples (25 g) were obtained from the rectum of 130 dairy cows and transferred to a 50 ml sterile polypropylene tube and transported directly on ice to the Dairy Epi Lab at the VMTRC, California. Fresh faecal samples were plated onto CHROMagar^™^ STEC medium (CHROMagar^™^, NJ, USA) selective for STEC isolation. Sterile cotton swabs soaked in 1X Tris-HCl were used to spread faecal samples onto agar. Plates were incubated for 18–24 h at 37 °C. After incubation, two presumptive STEC colonies were selected and picked per plate for pure colony isolation on bovine blood agar plates (UC Davis VM Biological Media Services) and incubated for 24 h at 37 °C. Two pure colonies per faecal sample were subjected to DNA extraction using Thermo Scientific GeneJET Genomic DNA Purification Kit (K0721, Thermo Scientific, USA). DNA extracts were isolated in 1.5 ml microcentrifuge tubes and stored at −20 °C until they were shipped to University College Dublin (UCD). Twenty dairy cows attending the UCD Veterinary Hospital were sampled for routine parasitological examination, and samples were split for culture. The sample of Irish cows was a convenience sample of dairy cows that presented to University Veterinary Hospital, UCD, that had faeces collected for routine screening as part of their case workup. Most cases were presented for routine conditions such as abomasal surgery. These were also sampled, and duplicates were plated on Sorbitol MacConkey Agar (Sigma-Aldrich, MO, USA). DNA was extracted from presumptive STEC cultures as described above. All DNA samples were stored at –20 °C until processing.

### PCR analysis of *stx1a* genes in UCD and Californian dairy herds

DNA samples isolated from STEC-infected dairy cows provided by UCD Veterinary Medicine School (*n*=14) and UC Davis, California (*n*=117), were initially screened for the presence of *stx1a* gene by endpoint PCR analysis. Standard PCR master mix per 15 µl reaction was prepared containing Dream Taq polymerase (7.5 µl), DNase-free sterile water (4.5 µl), 0.5 µM *stx1a* forward (CCGGACACATAGAAGGAAАСТС) and reverse primers (GGACAAGACTCTGTTCGT GTAG) (0.75 µl each) (Integrated DNA Technologies) and DNA template (1.5 µl) or water as a negative control. PCR reactions were performed with the following conditions: initial denaturation at 95 °C for 10 min, denaturation; 95 °C for 1 min, annealing; 53 °C for 1 min, elongation; 72 °C for 30 s, final elongation; 72 °C for 7 min for a total of 35 cycles with holding at 4 °C. Following PCR, amplified samples were mixed with Thermo Scientific^™^ TriTrack 6X DNA loading dye and run on 1% agarose gel supplemented with SYBR Safe DNA gel stain (5 µl) in 1X TAE at 150 V for 30 min.

### STEC strains for *in silico* analysis

The genomes of a selection of additional Irish STEC isolates (*n*=179) were examined *in silico* for the presence of the *adk*, *fkpA*, *gyrB*, *glnH*, *terD* and *yiaF* genes. These were STEC isolated from ovine recto-anal mucosal samples (*n*=167 [20]) and from bovine recto-anal mucosal samples (*n*=12 [21]). The ovine isolates represented a range of serotypes, while the bovine isolates were all *E. coli* O157. The accession numbers for the strains are PRJNA744868 and PRJNA91274, respectively.

### Real-time PCR primer and probe design

Forward and reverse primers and the corresponding TaqMan real-time PCR probe for each gene were designed using the Integrated DNA Technologies Inc. online PrimerQuest tool (https://www.idtdna.com/site/order/oligoentry and https://www.idtdna.com/site/order/qpcr/primetimeprobes) and using the *E. coli* O157:H7 reference sequence NC_002695 for each gene region of interest: *fkpA* (ECs4198), *glnH* (ECs0889), *yiaF* (ECs4439) and *terD* (ECs1355) (Table 1). The primers and probes and single- and multiplex compatibility checks were conducted to avoid extendable or non-extendable dimer formations by Integrated DNA Technologies Inc. (IDT, IA, USA). Once the primer and probe sets passed the appropriate internal checks for compatibility, individual probes were labelled with reporter molecules so that each of the four gene targets of interest was detected using four separate channels on the Roche LightCycler^™^ 96 Real-time polymerase chain reaction (RT-PCR) instrument (Roche Diagnostics, Germany). The excitation and emission wavelengths are detailed in Table 1. To minimize background and maximize sensitivity, double quenchers were used. The dark quencher, Iowa Black, was used for all dyes used in the multiplex reaction in combination with secondary quenchers, ZEN^™^ for FAM and HEX fluorophores and TAO^™^ for CY5 fluorophore.

**Table 1.** Primers and probes used in multiplex PCR assay*

Target genes	Primers/probe	Sequence	Amplicon size (bp)	Label used	λex/λem
*fkpA*	Forward	ACTGGGCATCAAACTGGATAA			
	Reverse	CAGAGTCTGTTCGATCTCTTGG	100	*FAM*	495/520
	Probe	FAM-TGATCGCTG-ZEN-GTGTTCAGGATGCA-IBFQ			
*glnH*	Forward	CGATTTCTCTGACGGCTACTAC			
	Reverse	TCTTCACCGCAACCACTTT	110	*HEX*	538/555
	Probe	HEX-AGCGGCCTG-ZEN-TTAGTGATGGTGAAA-IBFQ			
*yiaF*	Forward	GTTAGCCTGAGTGGGTGTTT			
	Reverse	CCGCTACGCATCACTGTATT	86	*TEX*	596/613
	Probe	TEX-AGAAGGCGATCAGCGTAAAGCGTT-IBRQ			
*terD*	Forward	AGGTATCCGGTGCGTTTATTC			
	Reverse	ACTCACCATTGTGGCGATAC	132	*CY5*	648/668
	Probe	CY5-TCCACTGAG-TAO-ACTGCCATGCTGTTC-IBRQ			

*IB, Iowa Black FQ/RQ.

### Real-time PCR amplification

Singleplex probe-based real-time PCR assays were designed to validate the efficiency of each primer pair and probe set by preparing reaction mixtures consisting of 25 ng DNA template (4 µl), forward and reverse primers (400 nM) and corresponding probe (200 nM, Table 1) with 10 µl 2X PrimeTime^™^ Gene Expression Master Mix (Integrated DNA Technologies, IDT) to a final volume of 20 µl. Optimal gene amplification conditions were as follows: preincubation at 95 °C for 10 min; two-step amplification of 30 cycles comprising denaturation (10 s at 95 °C) and primer annealing (30 s at 60 °C); and final cooling to 4 °C.

The detection of the presence of four antigen genes (*fkpA*, *glnH*, *terD* and *yiaF*) was performed using an amplification mixture containing 10 µl 2X PrimeTime Gene Expression Master Mix (Integrated DNA Technologies, IDT, IA, USA). A quadruplex hydrolysis probe-based real-time PCR reaction mixture consisted of 25 ng DNA template (4 µl), each set of forward and reverse primers at a concentration of 400 and 200 nM of corresponding probe sets (as outlined in Table 1) to a final volume of 20 µl. Gene amplification conditions required preincubation at 95 °C for 10 min and two-step amplification of 30 cycles consisting of denaturation (10 s at 95 °C) and primer annealing (30 s at 60 °C) with cooling to 4 °C. ‘No template’ controls using DNase- and RNase-free water were included in triplicate in each 96-plate assay to assess non-specific amplification or hetero-dimer formation.

Both single- and multiplex assays were run simultaneously to validate the multiplex efficiency. Singleplex assays involved individual primer and probe sets for each specific gene in separate reaction wells compared to multiplex assays, where all four primer and probe sets were applied to the same reaction well. Primer and probe efficiency were validated based on qualitative detection only and were considered valid if the point at which the reaction crossed the threshold within single- and multiplex assays differed between no more than two cycles. The presence of each antigen gene in quadruplex reactions was considered positive at a threshold (Cq) of less than 30 cycles. The genes were considered absent if amplification did not cross the threshold within 30 cycles. No amplification was observed in the controls.

### Bioinformatics analyses

Nextera adapter sequence removal and quality trimming of raw reads were checked using Trim Galore v0.6.1 (https://github.com/FelixKrueger/TrimGalore) with Cutadapt v1.18 [22] and FastQC v0.12.0 (https://www.bioinformatics.babraham.ac.uk/projects/fastqc/) incorporated, for which a Phred score of 33 and a minimum length of 75 bp were used. MultiQC v1.9 was further used for checking the quality of filtered raw reads and merging all reports as a single one [23]. The taxonomical assignment of filtered reads was done using Kraken2 v2.1.1 [24] by employing the minikraken2 database (https://benlangmead.github.io/aws-indexes/k2). Generated Kraken reports were compiled in one single report using an in-house pipeline ‘combine_kreports.py’ developed by Jennifer Lu (https://github.com/jenniferlu717).

Quality-filtered reads were assembled into contigs and scaffolds by SPAdes v3.15.3 [25], using default parameters, for which only scaffolds were retained for further analyses. A blastn alignment v2.8.1 [26] of scaffolds against an in-house database containing genes of interest (*fkpA*, *glnH*, *terD* and *yiaF*) together with other two housekeeping genes (*adk*, *gyrB*) [27] as internal positive controls was performed, employing an identity cut-off of 80%. The coverage percentage was calculated based on the ratio between alignment length and subject sequence length. The custom database was created using ‘makeblastdb’ containing *fkpA*, *glnH*, *terD *and *yiaF*, as well as *gyrB* and *adk* genes as internal positive controls. The blast reports were manually combined in one single report for further statistical analyses.

### Statistical analysis and data visualization

The data analysis was performed using R version 4.2.1 (https://cran.r-project.org/) and RStudio 2022.07.2 (https://posit.co/products/open-source/rstudio/), employing *dplyr* and *tidyverse* R packages.

## Results

### Examination of the presence of antigen genes in STEC-confirmed clinical paediatric isolates

**Fig. 1.**
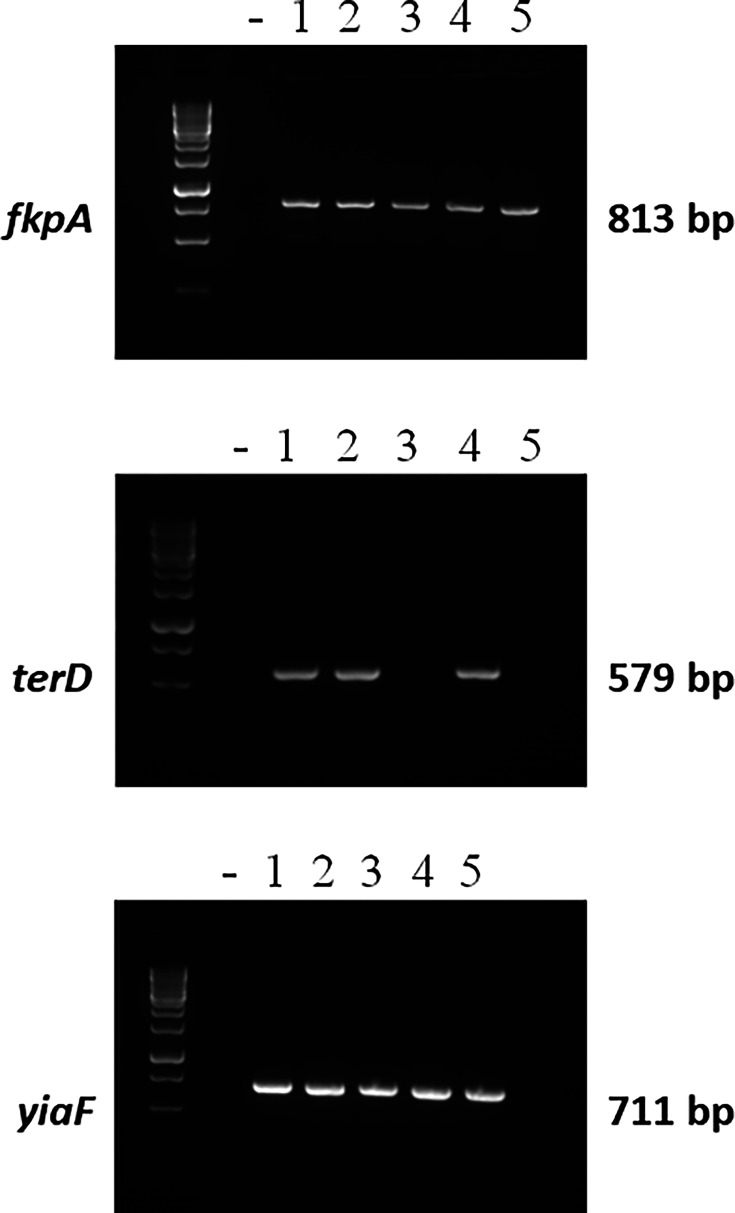
Presence of three genes of interest (*fkpA*, *terD* and *yiaF*) in STEC isolates from STEC-confirmed paediatric patients hospitalized for STEC infection. The presence of the genes was confirmed by colony PCR followed by visualization on 2% agarose gels.

Four antigen genes were chosen for this study: GlnH and FkpA were previously shown to be involved in attachment of the O157 prototype strain to HT-29 and Caco-2 cells [19], while YiaF and TerD were identified as being involved in attachment to HT29 cells only. As an initial step, we wanted to evaluate whether these genes were also present in human STEC clinical isolates. Five paediatric isolates from patients hospitalized for STEC infection were used in this initial study. We had previously shown that the *glnH* gene was present in all five paediatric isolates by PCR [19]. Similarly, *fkpA* and *yiaF* amplicons were both present in all five clinical paediatric STEC isolates (Fig. 1). In contrast, the *terD* gene was only amplified in three of the five isolates, suggesting a lower prevalence in this small sample.

### DNA quantitation

The low paediatric patient sample size, together with the apparently lower prevalence of the *terD* gene, led us to question how prevalent the four genes were in a wider population and also whether levels of prevalence would be different in two geographically distinct countries or regions, Ireland and California, USA. A total of 130 cows were selected at random from two separate pens of a large dairy herd (1,100 milking cows) in the San Joaquin Valley in California. Of 130 cows sampled, 117 faecal samples tested positive for STEC by culture. Of the 20 dairy cows sampled in Dublin, 14 tested positive for STEC by culture. The DNA extracted from the isolates ranged from 3 to 37 ng µl^−1^, with a median value of 12.2 ng µl^−1^, while the DNA extracted from the Dublin isolates ranged from 0.4 to 2.3 µg µl^−1^, with a median value of 1.2 µg µl^−1^. DNA concentration was standardized at 25 ng DNA/well for each sample screened for qPCR analysis. All STEC culture-positive samples were confirmed to be *stx1a* positive by PCR (Fig. S1).

### Multiplex real-time PCR validation

When primer and probe sets for the target genes were evaluated singly or in combination as a quadruplex assay on 96-well plates to validate the multiplex efficiency of each primer and probe, there was no difference in cycle threshold (Cq) variation between amplification of target genes in both single- and multiplex assays. The Cq between single- and multiplex assays for each primer/probe set only differed within an acceptable level of two cycles, highlighting that they were compatible in combination in a single reaction tube and that neither Cq nor specificity was affected when each primer/probe set was combined (Table 2 and Fig. S2).

**Table 2.** Cycle threshold variation (Cq) between single- and multiplex RT-PCR assays to validate primer and probe efficiency and compatibility

DNA sample	PCR reaction well	Average Cq mean		ΔCq singleplex versus multiplex
		*Singleplex*	*Multiplex*	
**Internal control *E. coli* O157:H7 NCTC12900**	*fkpA*FAM NCTC	14.88667	14.85	0.03667
	*glnH*HEX NCTC	14.46667	14.38667	0.08
	yiaFTEX NCTC	15.58667	15.14667	0.44
	terDCY5 NCTC	16.68333	16.48667	0.1966
**Test COW ID 132A +ve for all four genes**	*fkpA*FAM COW132	13.17	13.06333	0.10667
	*glnH*HEX COW132	15.60333	16.76	1.15667
	yiaFTEX COW132	13.67	13.30333	0.36667
	terDCY5 COW132	14.91333	14.54667	0.3666

### Antigen prevalence in a Californian dairy herd population

**Fig. 2.**
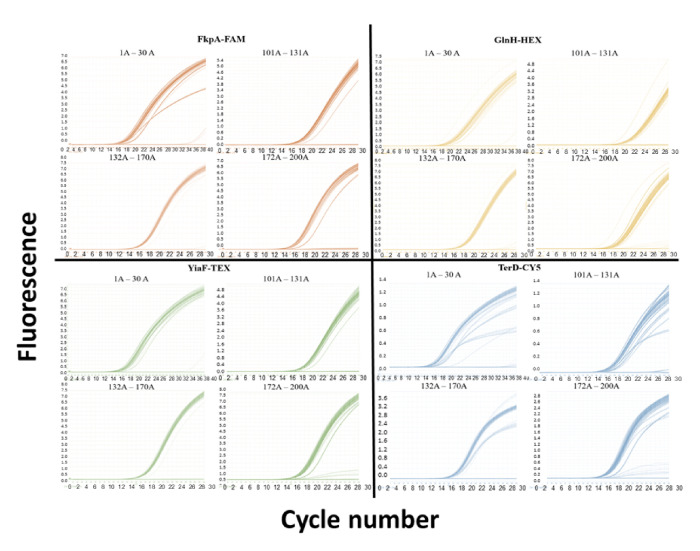
Amplification plots for each targeted gene in STEC-positive cow faeces from a Californian dairy herd. Rapid detection of antigen genes in 117 STEC DNA samples isolated from faeces shed from a Californian dairy herd by hydrolysis probe-based quadruplex RT-PCR using 4 individual fluorescent channels was confirmed by threshold crossing within 30 cycles. Failure to cross the threshold before 30 cycles or no amplification at 30 cycles was considered negative for any gene. Plate numbers correspond to the following samples: 1: 1–30A; 2: 101–131A; 3: 132–170A; and 4: 172–200A.

DNA extracts from STEC colonies isolated from 117 dairy cows were screened by multiplex real-time PCR to determine the prevalence of genes encoding for the 4 identified antigens: FkpA, GlnH, YiaF and TerD. All four antigen genes were highly prevalent among STEC DNA isolates in the dairy herd cohort, with 100% prevalence for *fkpA* and *yiaF* genes and >96% prevalence for *glnH* and *terD* genes, as depicted by amplification plots specific for each gene within individual PCR reaction wells (Fig. 2, Table 3).

**Table 3.** Comparison of prevalence (standard error) of individual antigen genes in dairy cows in California and Dublin. The prevalence of genes for the four selected antigens of interest in STEC samples isolated from faeces shed from cattle in a dairy herd population in California (*n*=117) and cows attending the UCD Vet. Hospital, Dublin (*n*=14)

Population	*fkpA*-FAM	*glnH*-HEX	*yiaF*-TEX	*terD*-CY5
**Californian herd prevalence**	117/117	114/117	117/117	113/117
	100% (0)	97.4% (1.46)	100% (0)	96.6% (1.68)
**Dublin sample prevalence**	13/14	13/14	13/14	1/14
	93% (6.89)	93% (6.89)	93% (6.89)	7% (6.89)

### Antigen gene prevalence in a subgroup of Dublin dairy cows

**Fig. 3.**
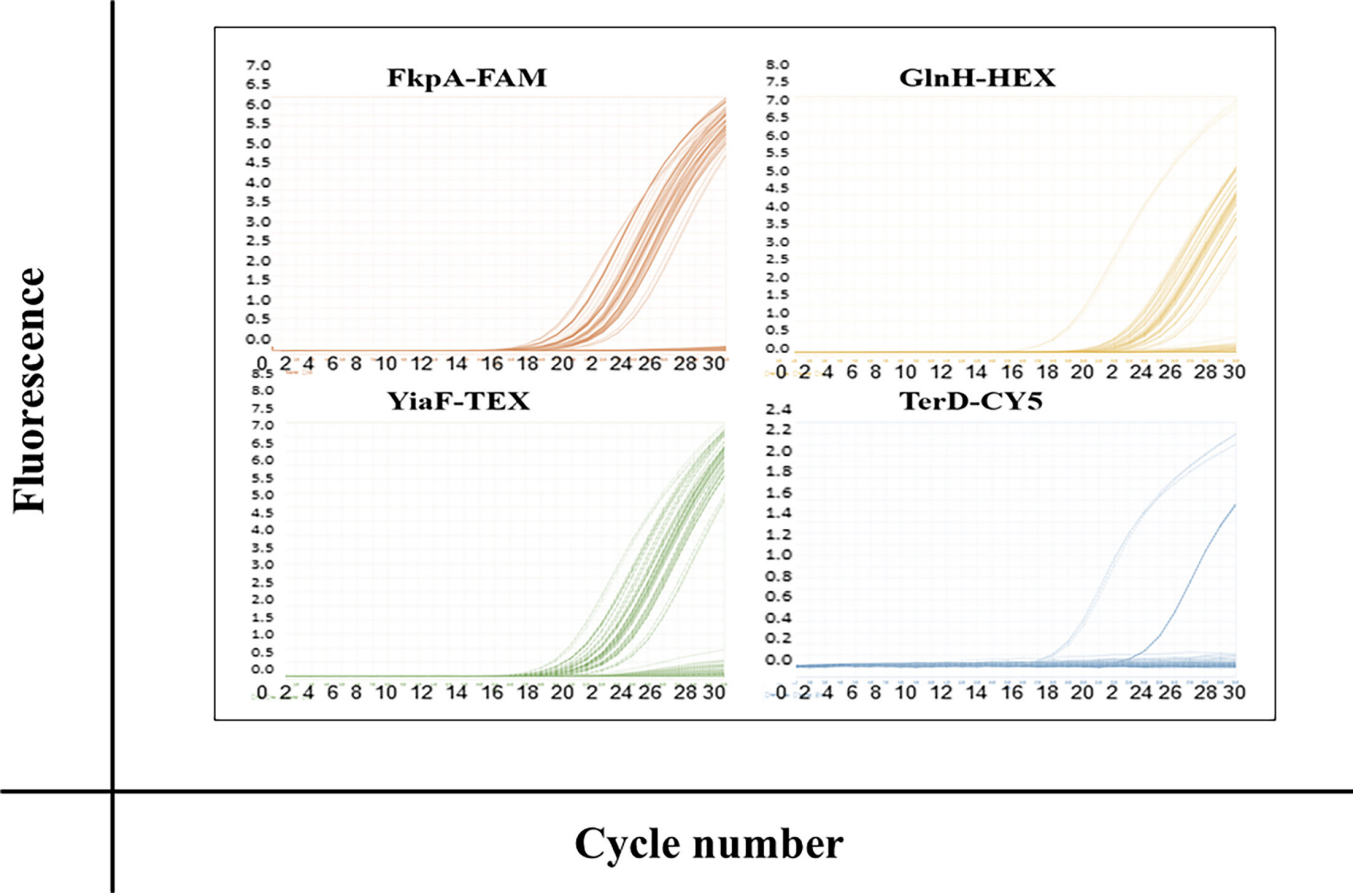
Amplification plots for each targeted gene using DNA isolated from STEC-positive cow faeces from dairy cows attending the Veterinary Hospital, UCD. STEC samples isolated from faeces shed from a Dublin dairy herd population. Rapid detection of antigen prevalence by hydrolysis probe-based quadruplex RT-PCR was confirmed by threshold crossing within 30 cycles. Failure to cross the threshold before 30 cycles or no amplification detected at 30 cycles was considered negative for a particular gene.

DNA samples isolated from 14 dairy cows provided by UCD Veterinary Medicine Hospital were screened as a preliminary study to investigate whether there were geographical differences in the presence of the four selected antigen genes between the two regions. Isolates screened from the 14 cows sampled at the UCD SVM showed that *fkpA*, *glnH* and *yiaF* were highly prevalent (93%). In contrast, the prevalence of the *terD* gene was low, with only 1 sample out of 14 isolates tested showing the presence of the *terD* gene (7%) (Fig. 3, Table 3).

### Identification of target genes in whole-genome sequence data of Irish bovine and ovine STEC isolates

Given the low prevalence of *terD* in the Irish dairy cow isolates, we sought to compare the presence of this gene in a broader repository which was available from bovine and ovine STEC isolate whole genome sequencing (WGS data). We also broadened the scope to ovine isolates as sheep are increasingly recognized as important contributors to dissemination of STEC [20,28]. The *gyrB* and *adk* genes were included as positive controls and were detected in all isolates. The *fkpA*, *glnH* and *yiaF* genes were also detected in all genomes examined. The *terD* gene was detected in all bovine isolates but in only 25.7% of the ovine isolates (Table 4).

**Table 4.** Detection of target genes in whole-genome sequence data of Irish bovine and ovine STEC isolates. The values in parentheses indicate the percentage of isolates in which the gene was detected and their standard errors

Gene	*fkpA*	*glnH*	*terD*	*yiaF*
Bovine isolates	12 (100%; 0)	12 (100%; 0)	12 (100%; 0)	12 (100%; 0)
Ovine isolates	167 (100%; 0)	167 (100%; 0)	43 (25.7%, 3.39)	167 (100%; 0)

Sheep harbour a wide variety of non-O157 STEC [20], and consequently, the impact of serotype was also examined. In total, isolates from 34 serotypes were examined, and of these, 15 had at least 1 isolate which was *terD* positive (Table 5). The dominant serotypes examined were O128:H2, of which 3 out of 33 isolates were *terD* positive, and O91:H14, where none of the 31 isolates examined encoded the *terD* gene. In contrast, 14 of 15 O146:H21 isolates examined encoded the *terD* gene, and 7 of 8 O100:H30 isolates encoded the *terD* gene.

**Table 5.** Sheep isolate serotypes examined and *terD* gene carriage

Ovine isolate serotype	No. of isolates	*ter* D+	% positive (SE)
O5:H9	1	1	100 (0)
O5:H19	4	0	0 (0)
O6:H10	13	0	0 (0)
O8:H19	3	0	0 (0)
O8:H9	2	1	50 (35.36)
O15:H27	2	1	50 (35.36)
O21:H14	2	0	0 (0)
O27:H7	1	0	0 (0)
O38:H26	1	1	100 (0)
O75:H8	5	0	0 (0)
O76:H19	6	0	0 (0)
O78:H4	6	0	0 (0)
O78/100:H4	1	0	0 (0)
O79:H14	1	1	100 (0)
O81:H21	1	0	0 (0)
O87:H16	5	0	0 (0)
O91:H14	31	0	0 (0)
O100:H4	1	0	0 (0)
O100:H30	8	7	87.5 (11.69)
O103:H2	1	1	100 (0)
O104:H7	1	0	0 (0)
O113:H4	1	0	0 (0)
O123:H10	1	0	0 (0)
O123/186:H10	2	0	0 (0)
O128:H2	33	3	9.1 (5.0)
O128:H21	1	1	100 (0)
O136:H20	1	0	0 (0)
O145:H28	1	1	100 (0)
O146:H21	15	14	93.3 (6.44)
O153:H5	1	0	0 (0)
O153/178:H7	2	1	50 (35.36)
O157:H7	1	1	100 (0)
O166:H28	10	7	70 (14.49)
O176:H4	2	2	100 (0)

## Discussion

We developed a high-throughput, probe-based multiplex real-time PCR approach to investigate antigen prevalence in a large population of ruminant samples and demonstrated that three antigen genes, *fkpA*, *glnH* and *yiaF*, are highly prevalent in distinct populations of bovine ruminants which highlights their potential breadth of coverage. The probe-based real-time PCR approach provided an enhanced level of specificity compared to using SYBR Green PCR and results in fewer false-positive amplifications [29]. In addition, it allowed processing of up to 30 STEC DNA samples in triplicate on one 96-well plate, with simultaneous detection of multiple genes in a single reaction well over a 1.5 h timeframe. Each probe was labelled with different distinguishable reporter dyes (FAM, HEX, TEX and CY5) which allowed amplification of four distinct gene sequences in the one reaction tube specific for antigen genes *fkpA*, *glnH*, *yiaF* and *terD*. The validation showed that the Cq variation was null (<2 cycles) between single- and multiplex assays for each gene; therefore, each primer/probe set was both efficient and compatible in combination in a single reaction well.

California and Ireland were chosen as examples of geographically distinct populations because Ireland is one of two leading countries in Europe in terms of prevalence of STEC, and STEC is also highly prevalent in California (4 cases per 100,000 people) and, importantly, the lead producer of leafy green vegetables associated with several US outbreaks. (cdph.ca.gov). All four antigen genes were highly prevalent among STEC DNA isolates obtained from dairy cows in two pen populations in the California herd, with 100% prevalence of genes for *fkpA* and *yiaF* antigens (117 out of 117), and >96% prevalence of antigen genes *glnH* (114 out of 117) and *terD* (113 out of 117). A smaller cohort of cows admitted to the UCD Veterinary Hospital in Dublin was compared to explore geographical differences between the study animals from Ireland and California. In this convenience sample, the prevalence for antigen genes *fkpA*, *glnH* and *yiaF* (13 out of 14) was comparable to those in the California herd. The high prevalence of antigen genes *fkpA*, *glnH* and *yiaF* among dairy herds and indeed in the ovine population is promising, as it indicates that should these antigens be protective *in vivo*, these may have the potential to offer broad protection against O157 and selected non-O157 STEC serotypes and could offer the same level of protection in geographically distinct regions. Such coverage would need to be confirmed in other populations elsewhere, including other countries and continents.

Variability in *terD* gene carriage in STEC isolates has been observed previously [30]; however, the extremely low prevalence of the *terD* antigen gene in the cow isolates (7%, 1 out of 14) and low prevalence in clinical isolates (60%, 3 out of 5) in Dublin, Ireland, compared to the high prevalence of this antigen among Californian isolates processed may not be indicative of a substantial geographical difference in *terD* prevalence. Indeed, this was not surprising considering the differences in isolation procedures used. The individual veterinary medicine laboratories in California and Ireland utilize different STEC selective agars as standard. Hence, the Californian bovine isolates that underwent PCR analysis were isolated using cefixime tellurite sorbitol MacConkey (CT-SMAC), which provides a selective pressure for the presence of the *terD* gene, while the standard STEC isolation in Ireland utilizes SMAC without tellurite and thereby introduced a bias in TerD prevalence at the different locations. Due to the bias in isolation media, further analysis was warranted. WGS data highlighted 100% prevalence of *terD* in Irish bovine isolates and in 25% of ovine isolates. The bovine isolates that underwent WGS analysis were isolated in a study that focused on the specific isolation of *E. coli* O157, and CT-SMAC was used to recover these [21]. In contrast, the ovine isolates were collated in a study that aimed to isolate a broader range of STEC serotypes, and therefore, the isolation media used was tryptone soy agar, followed by *stx* gene PCR screening [20], and the selective pressure for tellurite resistance is not present. Thus, the *terD* gene was not enriched in this latter cohort. This confirms that the differences in the *terD* prevalence in the three Irish datasets [real-time PCR (7%), bovine isolate WGS (100%) and ovine isolate WGS (25%)] are likely due to the isolation protocol used and illustrate the importance of the isolation medium and its components in *terD* screening, specifically.

A high prevalence of *terD* in STEC strains has been reported [31,33]. A study in a German cluster identified *terD* in 100% (80 out of 80) O104:H4 samples following an outbreak [31]. Similarly, the prevalence in a Nebraska study showed that 94.3% of STEC strains were *terD* positive, apart from O103 strains which returned a prevalence of 70% which correlated with K_2_TeO_3_ MIC values [32]. Rump *et al.* previously identified variations in the prevalence of *terD* gene between O157:H7 and O157:non-H7 STEC serotypes [33]. Specifically, *terD* was found in 96% O157:H7 isolates tested, consistent with the prevalence among STEC shed from the Californian dairy herd, but there was a significantly lower prevalence of 19% among O157:non-H7 isolates examined. A study on O26:H11 strains reported geographic consistency with 43 out of 47 strains being positive for *terD* across 11 countries from 4 continents, North and South America, Australasia and Europe. Previous studies have shown that *terD* in a DNA vaccine formulation elicited strong humoural and cell-mediated immune responses in immunized hybrid snakeheads and was highly protective with a relative per cent of survival of 83.14% against artificial challenge with *Nocardia seriolae*, a widespread chronic granulomatous disease in the aquatic environment [34]. This, together with our identification of TerD as an STEC adhesin, suggests its potential as an STEC vaccine antigen. However, in order for a vaccine to have global breadth of coverage, a consistently high prevalence is required. The lack of consistent expression of gene *terD*, which is associated with virulence of several pathogenic bacteria, including *E. coli* O157:H7 [33,35], limits its potential as a monovalent vaccine antigen. Intimin, a previously identified adhesin, has limited potential as a vaccine because antibody-mediated intimin protection was subtype specific with two of six intimin subtypes identified, β and γ, indicating that intimin may not protect broadly against infections caused by STEC strains expressing heterologous intimin subtypes [36,37]. That said, TerD may have potential in a multivalent vaccine.

A limitation of the current study is the cross-sectional nature of the sample collection, where a longitudinal study may be needed for estimating the risk of STEC shedding. Further, the study herds may not represent their locality or region; hence, further survey studies of dairy cows and other species that pose a risk for STEC infection in humans across multiple regions are necessary. This is particularly important when considering the relatively small convenience sample from cattle that presented to the UCD Veterinary Hospital, which necessitated additional *in silico* analysis across a broader sample. We have not evaluated the temporal variation or seasonal shedding dynamics in this study; however, this has been investigated by others at harvest and epidemiologically [20,21, 38]. The small sample size of the paediatric patient cohort limits the generalizability of the data. Moreover, it is acknowledged that we have only evaluated the presence of the gene in isolates rather than its expression or indeed immunogenicity in humans. However, in the context of future vaccine development, the presence of *fkpA* and *yiaF* antigen genes in all five paediatric faecal samples tested suggests that there is potential for these antigens as human vaccines. Moreover, the high level of prevalence associated with *fkpA*, *yiaF* and *glnH* genes in dairy cow and ovine populations indicates the potential for these antigens to be present in a broad range of STEC strains. In addition, *terD* shows variable prevalence which is masked by tellurite-containing selection media. Despite not having broad prevalence, *terD* antigen may still have potential as a protective multivalent vaccine candidate, enhancing coverage of *terD*-positive strains.

## Supplementary material

10.1099/jmm.0.002132 Uncited Supplementary Material 1.

## Abbreviations

CT-SMAC: cefixime tellurite sorbitol MacConkey
EU: European Union
HUS: haemolytic uraemic syndrome
RT-PCR: real time polymerase chain reaction
STEC: Shiga toxin-producing *Escherichia coli*
UCD: University College Dublin
WGS: whole genome sequencing
