# Supplementary material for: Prevalence of selected Shiga toxin-producing Escherichia coli vaccine antigen genes among two geographically distinct ruminant populations
Source: J Med Microbiol. 2026 Mar 19;75(3):002132. doi: 10.1099/jmm.0.002132 (PMC13002252; doi:10.1099/jmm.0.002132)
Supplement: Uncited Supplementary Material 1. [file jmm-75-02132-s001.pdf]

Prevalence of selected STEC vaccine antigen genes among two geographically distinct ruminant populations

Conor Quinn [1,2,] Rhys Bruce, Joanne Cosgrove, Laura Sala-Comorera[1], Niamh Martin[1], Catherine M. Burgess[3,] Elena-Alexandra Alexa[3], Catherine McAloon[4], Susanna Frost[5], Emmanuel Okello[6,7], Geraldine Duffy[3], Sharif S. Aly[6,7], Siobhán McClean[1,2]*

Supplemental Data

Table S1. Forward and reverse primers designed for selected antigen genes for PCR analysis

Target genes	Primers/ probes	Sequence	amplicon size (bp)
fkpA	Forward	CACCATGAAATCACTGTTTAAAGT	
	Reverse	TTATTTTTTAGCAGAATCTG	813
glnH	Forward	CACCATGAAGTCTGTATTAAAAGT	
	Reverse	TTATTTCGGTTCAGTACCGA	747
yiaF	Forward	CACCATGGCGACAGGAAAGTCCTG	
	Reverse	TTATTGGGTTGCAGTAACTG	711
terD	Forward	CACCATGAGTGTTTCTCTTTCCAA	
	Reverse	TCAGGACGCGTTAATGCCGT	579

Supplemental Figure 1

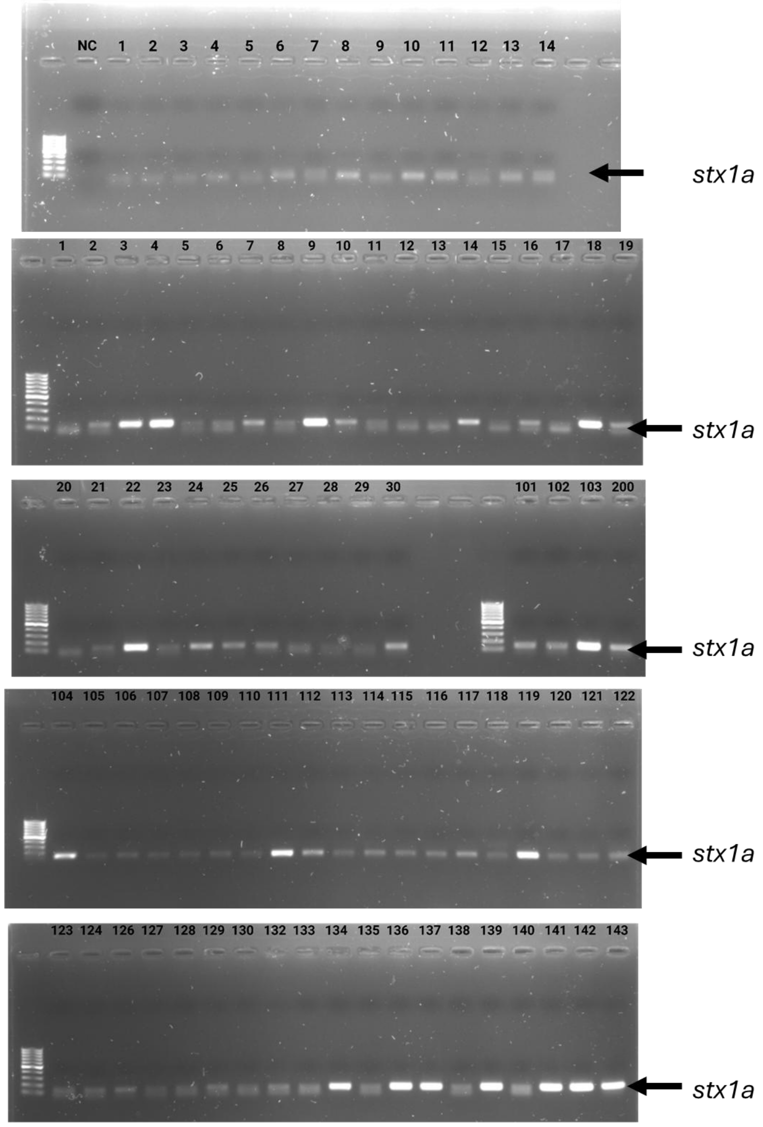

Figure S1. Presence of *stx1a* gene in bovine isolates from Dubin Ireland (top panel) and selection of Californian (lower panels) isolates by PCR.

Supplemental figure S2. Validation of single and multiplex primers and probes

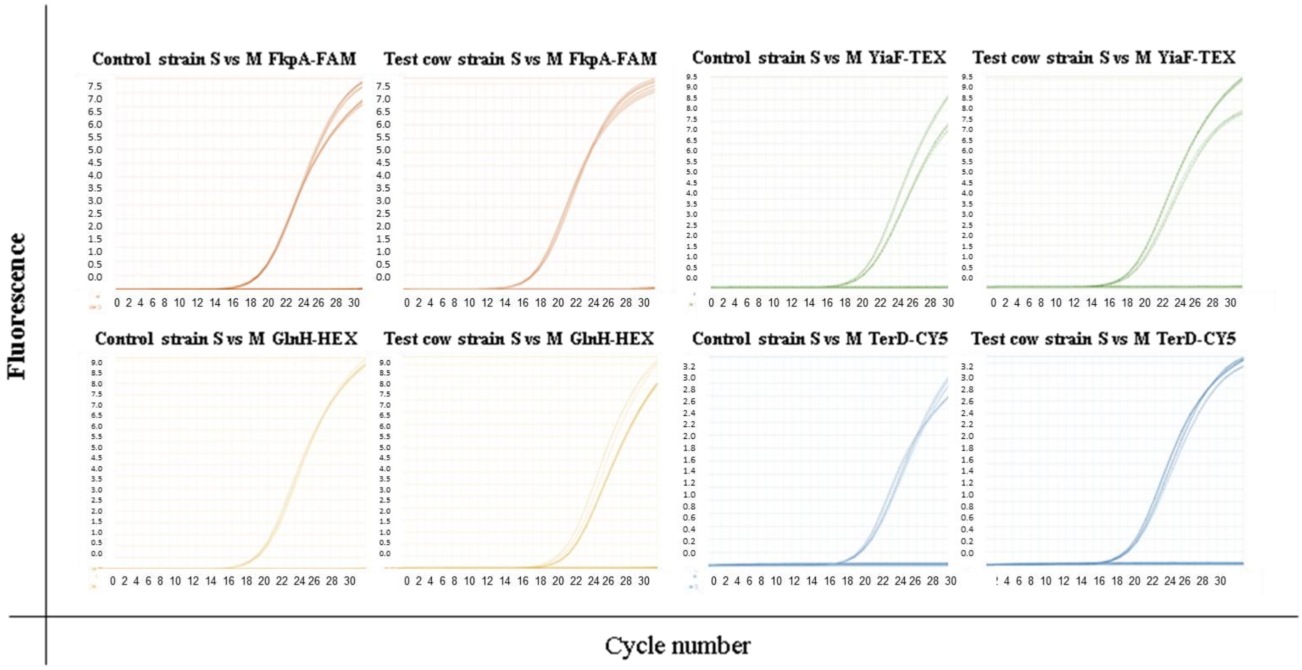

Validation of single and multiplex primers and probes. The presence of each antigen gene in single and multiplex reactions was confirmed by Cq within 30 cycles. Multiplex assay was validated by comparing the Cq determined for each antigen gene in single plex with the Cq identified for each gene using the quadruplex assay. Assays were considered valid with Cq values between single and multiplex assays varying by 2 cycles or less. Specific probes designed to detect individual genes based on simultaneous detection of each gene in the one reaction well at different wavelengths (channels).
